# Development of a recombinant human IgG1 monoclonal antibody against the TRBV5-1 segment of the T cell receptor for the treatment of mature T cell neoplasms

**DOI:** 10.3389/fimmu.2024.1520103

**Published:** 2024-12-17

**Authors:** Michele Pitaro, Giovanni Antonini, Alessandro Arcovito, Francesco Buccisano, Alfredo De Lauro, Maria Irno Consalvo, Valentina Gallo, Noah Giacon, Giuseppe Felice Mangiatordi, Maddalena Pacelli, Maria Teresa Pitaro, Fabio Polticelli, Matteo Sorrenti, Adriano Venditti

**Affiliations:** ^1^ INBB – Istituto Nazionale Biostrutture e Biosistemi, Rome, Italy; ^2^ Dipartimento di Scienze, Università di Roma Tre, Rome, Italy; ^3^ Dipartimento di Scienze Biotecnologiche di Base, Cliniche, Intensivologiche e Perioperatorie, Università Cattolica del Sacro Cuore, Rome, Italy; ^4^ Fondazione Policlinico Universitario Agostino Gemelli IRCCS, Rome, Italy; ^5^ Dipartimento di Biomedicina e Prevenzione, Università di Roma Tor Vergata, Rome, Italy; ^6^ Istituto di Cristallografia, Consiglio Nazionale delle Ricerche, Bari, Italy; ^7^ Tiber Biotech Srl, Rome, Italy

**Keywords:** T-cell neoplasms, T-cell receptor (TCR), human IgG1 monoclonal antibodies, phage display, surface plasmon resonance (SPR), flow cytometry, antibody-antigen docking

## Abstract

**Background:**

Mature T-cell neoplasms arise from the neoplastic transformation of a single T lymphocyte, and all cells in a neoplastic clone share the same V segment in the beta chain of the T-cell receptor (TCR). These segments may represent an innovative target for the development of targeted therapies.

**Methods:**

A specific V segment of the TCR beta chain (TRBV5-1) was analyzed using bioinformatic tools, identifying three potential antigenic peptides. One of these peptides, selected for synthesis, was used to screen a library of human single-chain variable fragments (scFv) through phage display. One fragment demonstrated high affinity and specificity for the antigen and was used to produce a human monoclonal antibody of the IgG1 class.

**Results:**

Surface plasmon resonance (SPR) studies confirmed the high affinity of the monoclonal antibody for the antigen in the nanomolar range. Flow cytometry analysis on patients’ samples demonstrated that the antibody, conjugated with a fluorochrome, selectively binds to tumor T lymphocytes expressing TRBV5-1, without binding to other lymphocytes or blood cell components.

**Conclusions:**

The development of fully human IgG1 monoclonal antibodies targeting specific V segments of the TCR beta chain represents a potential therapeutic option for patients with mature T-cell neoplasms.

## Introduction

T lymphocytes play a critical role in the immune response by identifying and eliminating infected or malignant cells (1). When these cells undergo malignant transformation, they can give rise to mature T-cell neoplasms (2), a relatively rare and aggressive group of cancers currently categorized by the WHO Classification of Haematolymphoid Tumours (3). Symptoms vary and may include lymphadenopathy, fever, night sweats, and, for cutaneous T-cell lymphomas, skin lesions (4).

Despite advancements in targeted therapies and immunotherapy, mature T-cell neoplasms remain a challenging clinical issue with limited treatment options, underscoring the need for innovative therapeutic strategies (5). These malignancies arise from the transformation of individual T cells, which express clonal T-cell receptors (TCRs) specific to the neoplastic clone (6). This feature presents a potential target for the development of monoclonal antibodies aimed at the variable (V) segments of TCRs expressed in malignant clones (7).

Several monoclonal antibodies have been developed in recent years for the treatment of specific forms of mature T-cell neoplasms (8). Alemtuzumab is a humanized IgG1 monoclonal antibody targeting the CD52 antigen, expressed on more than 95% of peripheral T and B lymphocytes (9). It is indicated for the treatment of fludarabine-refractory chronic lymphocytic leukemia (10) and has demonstrated efficacy in certain T-cell lymphomas, including cutaneous T-cell lymphoma (11) and peripheral T-cell lymphoma (12).

Brentuximab Vedotin is an antibody-drug conjugate (ADC) in which an anti-CD30 monoclonal antibody is conjugated with monomethyl auristatin E (MMAE), a cytotoxic agent (13). It is approved for the treatment of CD30-positive Hodgkin lymphoma (14) and relapsed or refractory anaplastic large-cell lymphoma (15). CD30 is expressed in a significant proportion of T-cell lymphomas, making this drug relevant for these malignancies (16).

Mogamulizumab is a humanized monoclonal antibody directed against the CCR4 receptor, which is expressed on certain malignant T cells (17). It has shown efficacy in the treatment of cutaneous T-cell lymphoma, particularly mycosis fungoides and Sézary syndrome (18).

Daratumumab, an anti-CD38 monoclonal antibody, is currently approved for multiple myeloma (19). However, preliminary studies have suggested potential efficacy in treating CD38-positive T-cell lymphomas, such as nasal-type NK/T-cell lymphoma (20).

Some monoclonal antibodies targeting the immune checkpoint PD-1, particularly Nivolumab (21) and Pembrolizumab (22), are currently under investigation for the treatment of refractory or relapsed T-cell lymphomas, with promising results in certain subtypes (23). The rationale behind these agents lies in the reactivation of the immune response against tumor cells (24).

Finally, bispecific antibodies capable of simultaneously binding an antigen on tumor cells and an antigen on healthy T cells (commonly CD3) are under investigation (25). These antibodies facilitate the activation of T cells against the tumor (26). This approach has shown promise in the treatment of B-cell lymphomas, where such antibodies promote the interaction between healthy T cells (via CD3) and tumor B cells (via CD20) (27). However, in the treatment of T-cell lymphomas, these antibodies appear to activate a fratricidal response, wherein healthy T lymphocytes and malignant T lymphocytes eliminate each other (28).

It is well known that the TCR consists of alpha and beta chains (or gamma and delta chains in γδ T cells), each with variable (V) and constant (C) regions (29), generated through V(D)J recombination to ensure broad antigen recognition (30). This diversity enables T cells to detect a wide array of antigens (31) but also poses challenges for targeted therapies due to the variability in TCR sequences (32). Targeting specific V segments, however, could enable precise therapeutic interventions against malignant T-cell clones (33).

Recent advances in phage display technology allow for the efficient screening of human single-chain variable fragments (scFvs) against specific antigens, enabling the production of fully human IgG1 monoclonal antibodies (34). This approach has the potential to deliver highly specific treatments for patients with mature T-cell neoplasms, offering a promising direction for future therapies.

## Materials and methods

### Objective

This study aimed to develop fully human monoclonal antibodies targeting the TRBV5-1 segment of the TCR beta chain. The affinity and specificity of these antibodies were assessed via bioinformatic analysis, surface plasmon resonance (SPR), and flow cytometry.

### Screening of human scFvs by phage display

Among the 68 V segments of the human TCR beta chain (35), TRBV5-1 was identified as a candidate target due to its significant expression in normal T lymphocytes (36) and potential presence in T-cell lymphomas. Antigenic peptides derived from the amino acid sequence of TRBV5-1 were synthesized by Proteogenix (Schiltigheim, France). The candidate peptides underwent analysis using two bioinformatic tools, Antigen Profiler Peptide (37) and AbDesigner (38), to assess antigenicity and structural compatibility, thereby confirming their suitability for the subsequent phage display process.

Proteogenix further conducted the screening of a proprietary library containing 5.37x10^10^ unique human scFv clones (LiAb-SFMAX™). This library was constructed from naive B lymphocytes isolated from 368 healthy donors across diverse ethnic groups (Caucasian, Arab, African, South American, and Asian), ensuring a broad representation of scFv diversity.

### Synthesis of r-hIgG1 monoclonal antibody

The selected scFv sequences were optimized for expression and used to produce the recombinant human IgG1 monoclonal antibody (r-hIgG1 mAb) by GenScript Biotech BV (Netherlands). These sequences were cloned and transfected into TurboCHO™ cells using a lentiviral vector system to ensure stable and high-yield expression. The antibody was then purified from the culture supernatant using protein A chromatography, which selectively binds the Fc region of IgG, yielding a highly concentrated product.

The purity and molecular integrity of the r-hIgG1 mAb were confirmed via SDS-PAGE, assessing molecular weight under reducing and non-reducing conditions, and by size-exclusion high-performance liquid chromatography (SEC-HPLC) to evaluate homogeneity and aggregate content.

### Synthesis of TCR

To assess affinity by SPR, a single-chain TCR (scTCR) expressing the TRBV5-1 segment was synthesized. Structural data from UNIPROT (39) (A0A578) and the Protein Data Bank (40) (PDB code 5BRZ) were referenced to ensure sequence accuracy and structural integrity. The variable regions of the alpha and beta chains were linked using a flexible linker peptide to preserve the TCR’s native structure and functional binding properties (41). A polyhistidine tag was added to facilitate purification and immobilization on the SPR sensor chip.

### Structural preparation and bioinformatics analysis

To evaluate the solvent accessibility and potential interaction of the immunogenic peptide with antibodies within the TCR, various bioinformatic techniques were employed.

The three-dimensional structure of the TCR (PDB code: 5BS0) was obtained from the Protein Data Bank. Due to possible imperfections in crystallographic structures, such as missing loops or unmapped atoms, Modeller was used to reconstruct the protein structure and minimize steric clashes (42).

For antibody-TCR docking, the protein structures were protonated using PDB2PQR to assign correct charges and accurately calculate electrostatic interactions (43). Energy minimization was performed with the “relax” utility of the Rosetta suite, guided by the REF2015 force field (44).

Docking with the six target antibodies was conducted using SnugDock, a module in Rosetta specific for antibody docking on protein structures (45). Ensemble docking was employed to account for the high flexibility of loop regions. A 300 ns molecular dynamics simulation of the TCR was executed using Amber (46), and the trajectory was clustered with cpptraj to generate a set of conformations (47).

Structural models of the six antibodies were obtained using AlphaFold Multimer, suitable for multimeric structures like antibodies composed of heavy (H) and light (L) chains (48). Models with the highest pLDDT scores were selected. Constant regions were removed, and a protein linker was added between the N-terminus and C-terminus of the two chains using Modeller to obtain the corresponding scFv.

Ensemble docking of the TCR conformations with each scFv was performed using SnugDock, allowing flexible modeling of the complementarity-determining regions (CDRs). This approach was crucial due to an unstructured amino acid loop in the interaction area with the target peptide, enabling accurate prediction of docking poses despite the presence of unstructured regions.

SnugDock generated a set of antibody-antigen structures ranked according to the REF2015 scoring function of Rosetta. For each scFv, the three structures with the best interaction energies were selected.

### Surface plasmon resonance

The interaction between the His-tagged scTCR (ligand) and the r-hIgG1 mAb (analyte) was measured using SPR on a Biacore X100 instrument (Biacore, Uppsala, Sweden). The His-tagged scTCR was immobilized on a nitrilotriacetic acid (NTA) sensor chip (Cytiva, Uppsala, Sweden) following the manufacturer’s protocol. The NTA surface was activated with Ni^2+^ ions to selectively bind the His-tagged scTCR, which could be removed during the regeneration phase by chelating the nickel ions with 0.35 M EDTA (pH 8.3; Sigma-Aldrich, St. Louis, MO, USA).

The Hys-tagged scTCR at a concentration of 44 μg/mL was injected over the sensor chip for 60 s, followed by a 10 s stabilization period. Analyte concentrations of 5, 2.5, 1.25, 0.625, and 0.3125 μM were tested. The SPR assay was performed at 25°C with a flow rate of 30 μL/min. The association phase was monitored for 180 s, and the dissociation phase for 600 s. The sensor chip surface was regenerated before each assay cycle by removing the bound His-tagged scTCR with EDTA and re-immobilizing fresh His-tagged ligand.

Kinetic parameters and the dissociation constant (K_D) were determined using Biacore X100 Evaluation Software (version 2.0.1 plus package). A 1:1 Langmuir binding model was applied to fit the experimental data, assuming a simple bimolecular interaction between the immobilized Hys-tagged scTCR and the analyte in solution.

### Flow cytometry

Flow cytometric analysis was performed on aliquots of fresh cells using a FACSLyric™ cytometer (BD Biosciences, San Jose, CA). Cells were adjusted to a concentration of 1 × 10^6^ cells/mL, and 100 µL of the suspension was placed into U-well tubes. Samples were incubated for 30 minutes at 4°C with a predefined combination of monoclonal antibodies to reliably identify neoplastic cells in Sézary syndrome: CD3 APC-H7, CD4 PE-Cy7, CD8 APC-R700, and CD45 V500-C (BD Biosciences).

Since monocytes express Fc receptors for immunoglobulins and could bind the r-hIgG1 mAb against the TRBV5-1 fragment via its constant region, a monoclonal antibody targeting CD14 (BV605, BD Biosciences) was included to identify and exclude monocytes. After lysing erythrocytes with isomolar NH_4_Cl buffer for 10 minutes, cells were washed three times with PBS containing 0.2% (w/v) azide and 0.2% (w/v) bovine serum albumin (BSA).

An amorphous gate encompassing all mononuclear cells while excluding erythrocytes and debris was set before acquisition. At least 1,000 CD3^+^ events per sample were acquired using a logical gate combining fluorescence/SSC and FSC/SSC plots. Data were analyzed using Infinicyte™ software (BD Cytognos, BD Switzerland Srl).

In the initial phase of the experiment, serial dilutions of the FITC-conjugated r-hIgG1 mAb were tested by adding 5 µL, 1 µL, and 0.5 µL of the antibody at a concentration of 1 mg/mL to the cell suspension. Results indicated that 0.5 µL of FITC-conjugated r-hIgG1 mAb provided an optimal signal, whereas larger volumes (1 µL and 5 µL) resulted in out-of-scale signals. This dilution was selected for the final experiments.

## Results

### Identification of TRBV5-1 antigenic peptides

The nucleotide and amino acid sequences of the TRBV5-1 segment are provided in **Table 1** and are available in the Protein Data Bank and IMGT database (14). Using 3D modeling and docking techniques with MHC class I molecules and the TCR alpha chain, three potential antigenic peptides were identified within TRBV5-1 (**Table 2**).

**Table 1:** Nucleotide and amino acid sequences of the TRBV5-1 segment.

Nucleotide sequence
GCCCAGTAAAGGCTGGAGTCACTCAAACTCCAAGATATCTGATCAAAACGAGAGGACAGCAAGTGACACTGAGCTGCTCCCCTATCTCTGGGCATAGGAGTGTATCCTGGTACCAACAGACCCCAGGACAGGGCCTTCAGTTCCTCTTTGAATACTTCAGTGAGACACAGAGAAACAAAGGAAACTTCCCTGGTCGATTCACAG
Amino acid sequence
MGSRLLCWVLLCLLGAGPVKAGVTQTPRYLIKTRGQQVTLSCSPISGHRSVSWYQQTPGQGLQFLFEYFSETQRNKGNFPGRFSGRQFSNSRSEMNVSTLELGDSALYLCASSL

**Table 2:** Amino acid sequences of the three antigenic peptides of the TRBV5-1 segment.

Antigenic peptide No. 1	Antigenic peptide No. 2	Antigenic peptide No. 3
AGVTQTPRYLIKTRGQQVTLSC	GRFSGRQFSNSRSEMNV	TLELGDSA

In red, the shorter version of peptide No. 1; in black and green, the two segments of antigenic peptide No. 2.

**Figure 1:**
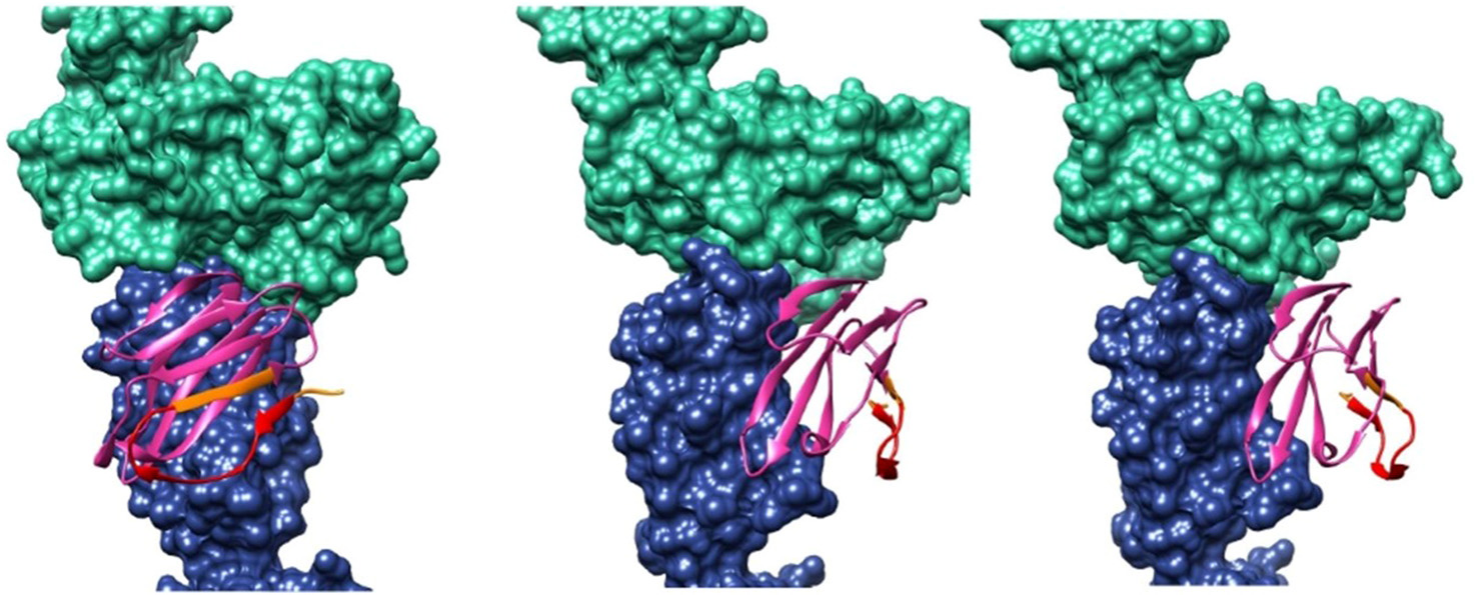
3D modeling and docking images showing the TCR alpha chain (in blue), the TRBV5-1 segment (in pink), and an HLA I/β2-microglobulin complex (in green). The antigenic peptide TQTPRYLIKTRGQQC is highlighted in red.

The first peptide, located near the N-terminal, is highly accessible to antibodies but is non-linear and thus challenging to synthesize. A shorter segment (highlighted in red in **Table 2**, **Figure 1**) may be preferable. The second peptide, positioned mid-segment, contains two regions (black and green in **Table 2**) with favorable properties for antibody development. The third peptide, closer to the C-terminal, also shows potential but contains residues susceptible to cleavage under acidic conditions.

Antigenic peptides for scFv screening were prioritized based on synthesis feasibility (**Table 3**). Peptide No. 1 and the first segment of Peptide No. 2 lack critical residues, making them ideal candidates. In contrast, the second segment of Peptide No. 2 and Peptide No. 3 contain amino acids like asparagine and methionine, which could hinder synthesis. **Table 4** shows the scores for each peptide as calculated by Antigen Profiler Peptide and AbDesigner. Peptide No. 1 (TQTPRYLIKTRGQQ) achieved high scores across tools, with an optimal length and no synthesis limitations, making it the best candidate for scFv screening.

**Table 3:** List of amino acids critical for peptide synthesis within each candidate peptide.

Peptide	Sequence	Critical amino acids
N. 1	TQTPRYLIKTRGQQ	None
N. 2 (segment 1)	GRFSGRQFS	None
N. 2 (segment 2)	NSRSEMNVS	Asparagine Methionine
N. 3	TLELGDSA	Cysteine Aspartic acid

**Table 4:** Scores returned by the three potential antigenic peptides.

Peptide	Sequence	Length	TM score	Ab-score
N. 1	TQTPRYLIKTRGQQ	14	2.5	5.8
N. 2 (segment 1)	GRFSGRQFS	9	2.5	5.8
N. 3	TLELGDSA	8	3.4	5.4

TM score computed by the Antigen Profiler Peptide tool; Ab-score computed by the AbDesigner tool.

### Selection of scFvs by phage display

The selection of scFvs using phage display started with conjugating the peptide TQTPRYLIKTRGQQ to three carrier proteins: BSA, OVA, and KLH. Panning cycles were conducted with tubes coated with the conjugated antigenic peptide, and non-specific phages recognizing only the carrier proteins were removed through washes. Phages binding the peptide were then eluted with Glycine-HCl, neutralized, and amplified in *Escherichia coli* TG1 culture for subsequent panning rounds.

To assess enrichment, a polyclonal ELISA was performed, coating multiwell plates with the conjugated peptide. After washing and blocking, phages from each panning cycle were incubated, followed by detection using an anti-phage HRP-conjugated antibody. The results indicated progressive enrichment from the first to the fourth cycle, with the fourth round yielding the highest specificity for the antigenic peptide.

Two rounds of monoclonal ELISA tests identified 18 positive clones expressing six sequences in the first test and 27 positive clones with five matching sequences in the second test. The consistent sequences across tests confirmed the reliability of the selection process.

A confirmatory monoclonal ELISA test further verified the binding specificity of the six clones, showing strong affinity for the TQTPRYLIKTRGQQ peptide (**Table 5**). Sequencing revealed that clones A2 and A5 displayed high identity (95%) in their VH regions, with only two amino acid differences in their CDR3 sequences. The other clones showed marked variability, with identity percentages not exceeding 71% (**Table 6**).

**Table 5:** Results of the confirmatory monoclonal ELISA test.

	Clone A2						Clone A5					
Phage dilution factor	Ag1	NC1	Ag2	NC2	Ag3	NC3	Ag1	NC1	Ag2	NC2	Ag3	NC3
1	0.95	0.24	4.3	0.22	2.46	0.32	0.88	0.12	4.09	0.28	2.73	0.34
3	0.91	0.19	4.04	0.04	1.85	0.18	0.37	0.03	3.8	0.05	2.53	0.15
9	0.35	0.02	3.84	0.02	1.28	0.08	0.11	0.02	3.74	0.02	1.55	0.07
Blank	0.02	0.01	0.01	0.01	0.02	0.02	0.01	0.01	0.02	0.01	0.02	0.02

	Clone A8						Clone A12					
Phage dilution factor	Ag1	NC1	Ag2	NC2	Ag3	NC3	Ag1	NC1	Ag2	NC2	Ag3	NC3
1	0.94	0.08	4.58	0.52	2.09	0.25	0.99	0.03	2.93	0.02	2.79	0.07
3	0.37	0.05	4.2	0.16	2.06	0.07	0.33	0.02	1.82	0.02	1.99	0.05
9	0.14	0.03	4.14	0.04	2.00	0.07	0.12	0.02	0.78	0.02	1.22	0.03
Blank	0.02	0.03	0.03	0.03	0.02	0.02	0.02	0.02	0.02	0.02	0.03	0.02

	Clone C10						Clone D5					
Phage dilution factor	Ag1	NC1	Ag2	NC2	Ag3	NC3	Ag1	NC1	Ag2	NC2	Ag3	NC3
1	1.19	0.04	3.83	0.04	2.11	0.07	0.75	0.04	4.03	0.17	2.38	0.24
3	0.75	0.02	3.55	0.02	1.72	0.05	0.32	0.04	3.56	0.09	1.90	0.06
9	0.42	0.02	3.04	0.03	1.62	0.04	0.13	0.05	3.5	0.07	1.67	0.05
Blank	0.02	0.03	0.02	0.04	0.02	0.03	0.04	0.06	0.06	0.06	0.03	0.04

Ag1, peptide alone; Ag2, peptide-BSA; Ag3, peptide-biotin; NC1, PBS; NC2, 10% BSA-PBS; NC3: PBS-streptavidin.

**Table 6:** Amino acid sequences of the six scFvs identified by phage display.

VH chain				
	CDR1	CDR2	CDR3	
**A2**	GDSVSSNSAA	TYYRSKWYN	ARDQAAAEVYFDY	
**A5**	GDSVSSNSAA	TYYRSKWYN	ARDQAAAEVYFDY	
**A8**	GYTFSSFD	MNPDTGNT	ARDAPRNGRGMDV	
**A12**	GGSFSGYY	INHSGST	ARWDLDY	
**C10**	GGTFSSYA	IIPIFGTA	AREPLRGYSGYDFYYYGMDV	
**D5**	GYYFNGYD	MNPNSGNR	ARERVSSGLDF	
	FR1	FR2	FR3	FR4
**A2**	QVQLQQSGPGLVKPSQTLSLTCAIS	WNWIRQSPSRGLEWLGR	DYAVSVKSRITINPDTSKNQFSLQLNSVTPEDTAVYYC	WGQGTLVTVSS
**A5**	QVQLQQSGPGLVKPSQTLSLTCAIS	WNWIRQSPSRGLEWLGR	DYAVSVKSRITINPDTSKNQFSLQLNSVTPEDTAVYYC	WGQGTLVTVSS
**A8**	QVQLVQSGPEMKKAGASVRVSCKGS	INWVRQVPGQGLEWMGW	GLAQKFQGRVTMTRDTSIRTAYMELRSLRSDDTAVYYC	WGQGTMVTVSS
**A12**	QVQLQQWGAGLLKPSETLSLTCAVY	WSWIRQPPGKGLEWIGE	NYNPSLKSRVTISVDTSKNQFSLKLSSVTAADTAVYYC	WGQGTLVTVSS
**C10**	EVQLVQSGAEVKKPGSSVKVSCKAS	ISWVRQAPGQGLEWMGG	NYAQKFQGRVTITADKSTSTAYMELSSLRSEDTAVYYC	WGQGTMVTVSS
**D5**	QVQLVQSGAEVKKPGASVKVSCKPS	ITWVRQAIGQGLEWMGW	DYAHQFQGRLTMTWDTSLSTAYLELNNLQSDDTAIYYC	WGQGTLVTVSS

VL chain				
	CDR1	CDR2	CDR3	
**A2**	SGSIASNY	EDN	QSYDNRNHWV	
**A5**	SGSIASNY	EDN	QSYDSSNHWV	
**A8**	ALPKQY	QDT	QSADSSGTYPV	
**A12**	QSLVHSDGNTY	QVS	MQGTHWPPIT	
**C10**	GGSIASNY	EDK	QSADSTSTYV	
**D5**	ALPKQY	KDS	QSADSSGTYVV	
	FR1	FR2	FR3	FR4
**A2**	NFMLTQPHSVSESPGKTVTISCTRS	LQWYQQRPGSAPTTVIY	QRPSGVPDRFSGSIDSSSNSASLTISGLKTEDEADYYC	FGGGTQLTVL
**A5**	NFMLTQPHSVSESPGKTVTISCTRS	VQWYQQRPGSSPTTVIY	QRPSGVPDRFSGSIDSSSNSASLTISGLKTEDEADYYC	FGGGTKVTVL
**A8**	SYELTQPPSVSVSPGQTARITCSGD	AYWYQQKPGQAPVLVIY	KRPSGIPERFSGSSSGTTVTLTISGVQAEDEADYYC	FGGGTKLTVL
**A12**	EIVLTQSPLSLPVTLGQSASISCRSS	LNWFQQRPGQSPRRLIY	KRDSGVPDRFSGSGSGTDFTLRISRVEAEDVGFYYC	FGQGTRLEIK
**C10**	SYELTQPHSVSGSPGETVTISCAGS	VQWYQQRPGSAPTTLIY	VRPSGVPDRFSGSIDYSSDSASLTISGLKTEDEADYYC	FGTGTKVTVL
**D5**	SYELTQPPSVSVSPGQTARITCSGD	AYWYQQKPGQAPVLLIY	ERPSGIPERFSGSSSGTTVTLTISGVQAEDEADYYC	FGGGTQLTVL

The codes in bold refer to the six scFvs.

### Selection and ranking of scFvs

The TCR structure from the Protein Data Bank (code: 5BS0) was modified by retaining only the constant and variable regions of the alpha and beta chains. The resulting structure underwent preparation using Modeller, PDB2PQR, and Rosetta, where hydrogen atoms, missing loops, and bond orders were added, and energy minimization was applied at pH 7.4.

To assess how the beta chain constant region’s loop might impact scFv binding, the antigen structure was subjected to a 300 ns molecular dynamics simulation in Amber. Clustering based on RMSD revealed three main conformations, representing 78% of the total population, for use in ensemble docking.

The six scFvs were modeled from primary structures using AlphaFold, then prepared as scFv constructs by linking the heavy (H) and light (L) chains in Modeller. Each scFv was optimized using PDB2PQR and Rosetta, and local ensemble docking was performed with SnugDock, targeting the designed antigenic region. The best interface energy complexes for each scFv were visually inspected. The docking analysis revealed that scFv C10 displayed inconsistent binding, preferring a region outside the designed target area. Based on this finding, C10 was excluded as a potential lead candidate.

**Table 7** ranks the five remaining scFvs by predicted affinity and notes possible interactions with the TCR beta chain constant region. Clones D5 and A12 emerged as top candidates, with D5 showing the highest predicted binding affinity and A12 demonstrating the greatest selectivity. A12 was ultimately selected as the lead scFv due to its superior binding selectivity, minimizing off-target effects.

**Table 7:** Ranking of scFvs based on interface energy score and interaction with TCR beta chain constant region.

Ranking Position	scFv (Interface Energy Score)	Number of TCR beta chain constant region interacting residues
1	d5 (-44.497 REU)	24
2	a12 (-37.749 REU)	6
3	a8 (-36.748 REU)	20
4	a2 (-33.626 REU)	18
5	a5 (-33.345 REU)	21

### Surface plasmon resonance analysis

**Figure 2:**
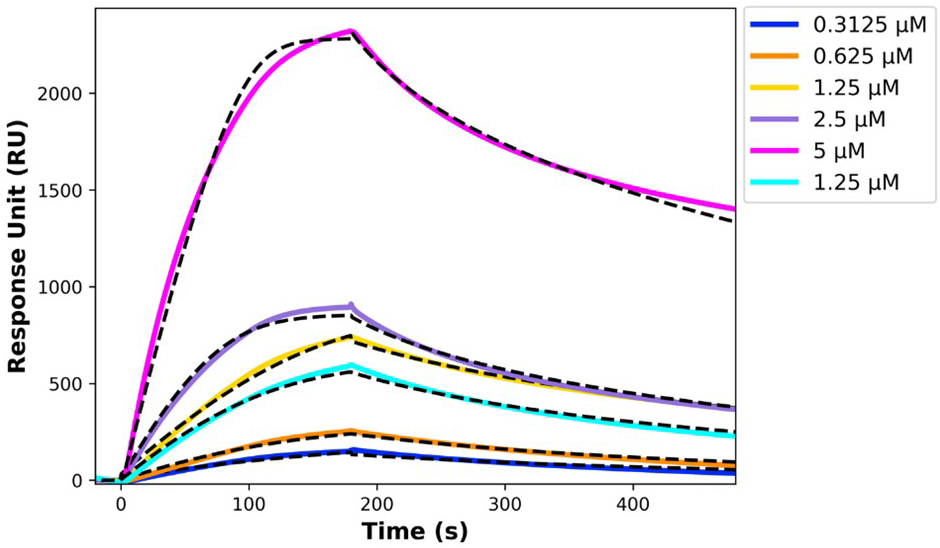
SPR sensorgrams showing the interaction between immobilized His-tagged single chain TCR and r-hIgG1 mAb, with black dashed lines representing the fitting curves. The TCR was immobilized on an NTA chip, and the r-hIgG1 mAb was injected at concentrations ranging from 5 µM to 0.3125 µM.

The kinetic parameters from the SPR experiment indicated a strong and specific interaction between the analyte (r-hIgG1 mAb) and the ligand (His-tagged scTCR), with a dissociation constant (KD) of 300 ± 13 nM, suggesting a moderate to high binding affinity. The SPR sensorgrams (**Figure 2**) were fitted using a global fitting procedure based on a 1:1 bimolecular interaction model, which assumes each ligand molecule binds to a single analyte molecule independently. This model allowed for precise measurement of interaction kinetics, providing a clear depiction of the binding behavior.

The kinetic rate constants, summarized in **Table 8**, offer deeper insights into the binding dynamics. The association rate constant (kon) and dissociation rate constant (koff) reflect a stable and dynamic interaction, where the ligand-analyte complex forms and dissociates predictably. This kinetic stability is favorable for potential therapeutic applications, as it indicates a reliable binding interaction.

**Table 8:** Kinetic rate constants.

kon (M^-1^s^-1^)	koff (s^-1^)
(1.98 ± 0.05) x 10^4^	(59.6 ± 1.1) x 10^-4^

### Flow cytometry analysis

Flow cytometry was performed on mononuclear cells isolated from peripheral blood samples (PBMCs) of two patients with mature T-cell neoplasm (Sézary syndrome). One patient expressed the TRBV5-1 segment in the variable region of the TCR beta chain, while the other expressed a different segment, TRBV6-5.

**Figure 3:**
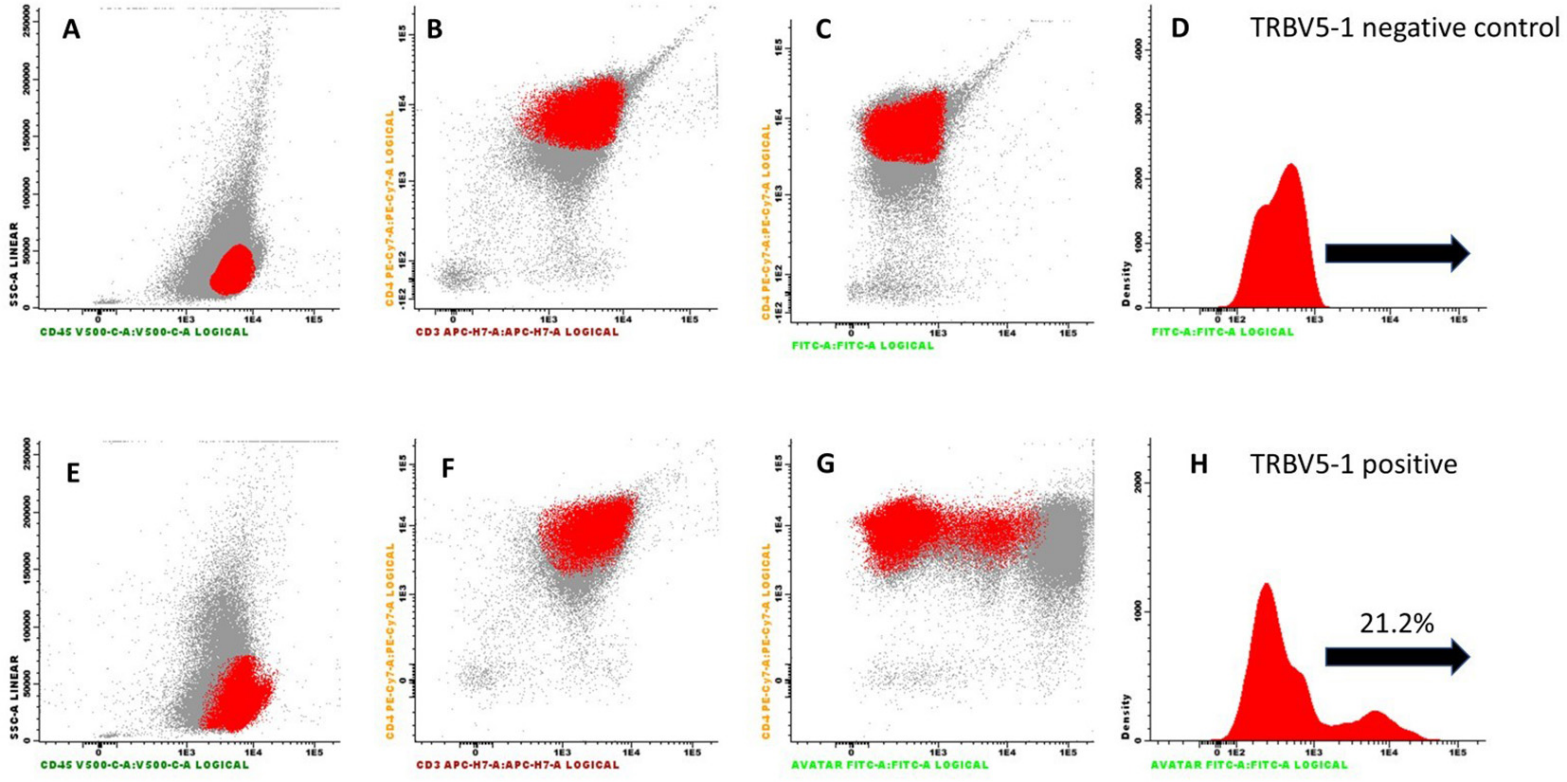
Flow cytometry results from PBMCs obtained from a patient with Sezary syndrome expressing the TRBV5-1 segment. Cells were pre-incubated with a complete panel of antibodies (CD3, CD4, CD8, CD45), along with an anti-CD14 antibody to identify monocytes. Panels **(A–C)** show the cell population of interest highlighted in red as dot plots. In panel **(D)**, the same population is represented as a histogram. Panels **(E–G)** refer to the test conducted with the addition of the FITC-conjugated r-hIgG1 mAb [with the same population represented as a histogram in panel **(H)**]. The test highlights the binding of the mAb to 21% of the cell population under investigation.

The optimal concentration of FITC-conjugated r-hIgG1 mAb was determined to be 0.5 mL. Two parallel tests were then conducted using the Fluorescence Minus One (FMO) technique to assess binding specificity. The FMO control compared the complete panel of monoclonal antibodies without r-hIgG1 mAb against the panel with the addition of r-hIgG1 mAb, to clearly identify the specific fluorescence signal due to r-hIgG1 mAb (**Figure 3**).

In the first test, conducted on PBMCs from the patient expressing the TRBV5-1 segment, the r-hIgG1 mAb stained 21% of the tested cell population, indicating specific binding to cells expressing the TRBV5-1 segment. In contrast, in the second test on PBMCs from the patient expressing the TRBV6-5 segment, r-hIgG1 mAb did not bind to the cell population (data not shown).

These results, consistent with previous SPR observations, confirmed that the r-hIgG1 mAb exhibits high specificity for the TRBV5-1 segment in the variable region of the TCR beta chain, without cross-reactivity with other TCR segments.

## Discussion

The development of a r-hIgG1 monoclonal antibody targeting the TRBV5-1 segment of the TCR represents a novel and targeted therapeutic strategy for treating mature T-cell neoplasms in which neoplastic clones express this specific segment.

Three-dimensional modeling and molecular docking identified three potential antigenic peptides within the TRBV5-1 segment, with TQTPRYLIKTRGQQ emerging as the ideal candidate based on its structural properties and favorable bioinformatic scores. Using phage display, six human scFvs were isolated, and clone A12 was selected as the lead due to its superior binding selectivity and favorable energy profile.

SPR analysis confirmed a strong interaction between the r-hIgG1 mAb and a scTCR containing the TRBV5-1 segment, with a dissociation constant of 300 ± 13 nM. The 1:1 kinetic model obtained from SPR supports a direct and specific interaction, reinforcing the potential of this antibody as a targeted therapeutic agent.

Flow cytometry further demonstrated the antibody’s specificity, showing successful binding to T cells expressing the TRBV5-1 segment. Notably, 21% of PBMCs from a patient with Sézary syndrome expressing the TRBV5-1 segment were stained by the FITC-conjugated antibody, while no binding was observed in cells from a patient expressing the TRBV6-5 segment. This high specificity underscores the antibody’s potential as both a diagnostic and therapeutic agent for targeting specific T-cell clones in mature T-cell neoplasms. This selectivity is crucial, as it allows the antibody to specifically target neoplastic T cells without affecting healthy cells, thereby potentially reducing adverse side effects commonly associated with therapies of lower specificity.

A broader implication of this approach is the opportunity to develop a set of fully human monoclonal antibodies against various V segments of the TCR beta chain, particularly those more frequently expressed in mature T-cell neoplasms. Monoclonal antibodies could offer a faster and more cost-effective therapeutic alternative to CAR-T therapies, which, while effective, are complex, time-consuming, and costly to produce (49, 50).

However, there are potential limitations to this therapeutic approach. Neoplastic clones might emerge with different TCRs, requiring adjustments in therapy (51). In this case, the use of a secondary monoclonal antibody targeting the V segment expressed by the new clone could help maintain therapeutic efficacy. Another limitation could involve the downregulation or loss of TCR expression in neoplastic T lymphocytes, reducing the effectiveness of a TCR-targeted antibody (52). This challenge could be addressed by combining the monoclonal antibody against the TCR beta chain with a second therapy targeting a different antigen, such as Mogamulizumab, which has shown efficacy in various T-cell neoplasms.

Future efforts will focus on increasing the therapeutic efficacy of our r-hIgG1 antibody prior to testing it in preclinical studies on animal models and subsequently in clinical trials in humans. Optimization of monoclonal antibodies aims to enhance Fc-dependent immune effector mechanisms, such as complement-dependent cytotoxicity (53) (CDC), antibody-dependent cell-mediated phagocytosis (54) (ADCP), and antibody-dependent cell-mediated cytotoxicity (55) (ADCC).

Complement activation in tumor cells, including T-cell lymphomas, is often hindered by the expression of complement regulatory proteins (CRPs) like CD46, CD55, and CD59 (56). In contrast, the effectiveness of ADCP in treating hematologic malignancies may be limited by the reduced phagocytic capacity of tissue macrophages, their restricted access to antibody-bound tumor cells, reduced antibody levels in lymphoid tissues, antigen loss through trogocytosis (57), and intrinsic resistance in certain tumor cell subpopulations (58).

ADCC, however, is emerging as a critical factor in the development of therapeutic monoclonal antibodies (59). Two primary approaches are commonly employed to enhance ADCC: modification of the Fc region and optimization of glycosylation patterns. Modifying the Fc region can significantly increase the antibody’s affinity for the FcγRIIIa (CD16) receptor on NK cells. A common approach is isotype engineering, with specific IgG variants, particularly IgG1, favored for their natural affinity for CD16, making them highly effective in inducing a cytotoxic response (60). This rationale guided our choice to produce an IgG1 monoclonal antibody from the scFv selected by phage display. Alternative approaches include introducing specific mutations, such as S239D/I332E (61), which can improve the antibody’s binding capacity to effector cells, though it remains unclear whether these mutations may increase immunogenicity.

Optimizing glycosylation further enhances ADCC (62). For example, removing fucose from the Fc region’s glycan structures, a process known as defucosylation, significantly improves the antibody’s affinity for CD16a on NK cells and is one of the most effective modifications in antibody development (63). This change not only strengthens direct tumor cell killing but also heightens immune activation through cytokine release and the recruitment of additional immune cells, creating a sustained anti-tumor response. Existing defucosylated antibodies, such as Obinutuzumab (64) and Mogamulizumab, highlight the effectiveness of this approach in onco-hematological therapies, suggesting that similar modifications could significantly boost the anti-tumor activity of our antibody. Defucosylated monoclonal antibodies can be produced at scale in Chinese hamster ovary (CHO) cells with gene knockouts for FUT8 or other glycosylation-related enzymes, making large-scale production feasible (65, 66).

## Conclusions

Our findings underscore the potential of a fully human, recombinant IgG1 monoclonal antibody targeting the TRBV5-1 segment as a targeted therapeutic for a subset of mature T-cell neoplasms. The antibody’s high specificity for the TRBV5-1 segment suggests that it could selectively eliminate neoplastic T cells in patients while minimizing off-target effects, thereby improving patient outcomes. This approach offers a promising pathway for patients with mature T-cell neoplasms where TRBV5-1 expression is a defining feature.

Looking ahead, advancing this antibody into preclinical efficacy studies and, subsequently, into human clinical trials will be essential to verify its therapeutic potential. Furthermore, developing a series of antibodies targeting the most frequently expressed V segments in T-cell neoplasms could provide a broader range of options for treating these rare and severe diseases. Unlike CAR-T cell therapies, this series of antibodies could allow for greater control over dosage and treatment combinations, offering a viable, cost-effective, and flexible solution for addressing this subset of rare and orphan hematological malignancies.

## Data Availability

The structures of the antibody-TCR complexes described in the manuscript have been made publicly available in Zenodo repository: https://doi.org/10.5281/zenodo.14258991.
